# The role of the comprehensive complication index in the prediction of tumor-related death in transplanted patients with hepatocellular carcinoma

**DOI:** 10.1007/s13304-025-02101-8

**Published:** 2025-02-10

**Authors:** Quirino Lai, Fabio Melandro, Alessandro Vitale, Davide Ghinolfi, Laurent Coubeau, Riccardo Pravisani, Greg Nowak, Federico Mocchegiani, Marco Vivarelli, Massimo Rossi, Bo-Göran Ericzon, Umberto Baccarani, Paolo De Simone, Umberto Cillo, Jan Lerut

**Affiliations:** 1 Organ Transplantation Unit, Department of General and Specialty Surgery, Sapienza University of Rome, AOU Policlinico Umberto I, Viale del Policlinico 155, 00161 Rome, Italy; 2 University of Padua, Padua, Italy; 3 University of Pisa, Pisa, Italy; 4 Université Catholique de Louvain, Brussels, Belgium; 5 Department of Medicine, University of Udine, Udine, Italy; 6 Karolinska University Hospital Huddinge, Solna, Sweden; 7 Polytechnic University of Marche, Ancona, Italy

**Keywords:** CCI, Mortality, HCC recurrence, Prognostic models, Survival rates

## Abstract

Liver transplantation (LT) is the primary treatment for selected patients with hepatocellular carcinoma (HCC). However, HCC-related mortality post-LT remains a significant concern, with up to 10% of cases reported in international series. Identifying risk factors for adverse clinical outcomes is essential. We hypothesized that post-LT HCC-related mortality rates are higher in patients with a high (≥ 42) Comprehensive Complication Index (CCI) calculated at discharge. This study aims to compare post-LT HCC-related mortality rates between two groups of patients with high versus low CCI following LT for HCC. This study included data from seven collaborative European centers. A cohort of 1121 HCC patients transplanted between 2005 and 2019, surviving more than six months post-LT, was analyzed retrospectively. Patients were divided into two groups based on the CCI at discharge: Low-CCI Group (*n* = 942, 84.0%) and High-CCI Group (*n* = 179, 16.0%). An inverse probability of treatment weighting (IPTW) approach was applied for analysis. In the post-IPTW cohort, four multivariable logistic regression models with mixed effects identified independent risk factors for HCC-related death, overall death, recurrence, and early recurrence. A CCI score of ≥ 42 emerged as an independent risk factor across all models. Specifically, CCI ≥ 42 was associated with increased odds of HCC-related death (OR = 3.35; *P* < 0.0001), overall death (OR = 2.63; *P* < 0.0001), overall recurrence (OR = 2.09; *P* = 0.001), and early recurrence (OR = 1.88; *P* = 0.02). A CCI score at discharge should be considered a critical factor for recurrence and HCC-related mortality risk. Incorporating CCI into standard post-LT predictive models may enhance prognostic accuracy for adverse HCC outcomes.

**Supplementary Information:**

The online version contains supplementary material available at 10.1007/s13304-025-02101-8.

## Introduction

Liver transplantation (LT) is the preferred treatment option for both acute and chronic liver failure, as well as primary liver malignancies like hepatocellular carcinoma (HCC) within well-established morpho-volumetric criteria [1]. In recent years, expanded criteria have integrated tumor markers (e.g., alpha-fetoprotein [AFP], protein induced by vitamin K absence or antagonist-II [PIVKA II]), growth rates, response rates to locoregional treatments, and artificial intelligence (AI) tools to improve post-LT outcomes and patient selection [2–4]. Despite these advancements, recurrence occurs in at least 10–15% of patients.

Over the past decades, several prognostic models have been developed to estimate HCC recurrence risk post-LT. Among these, the recently published RETREAT score combines pre-LT variables (e.g., AFP) and histological data (such as the number and size of active nodes and microvascular invasion) to predict post-LT HCC recurrence risk, showing greater accuracy than the Milan and San Francisco Criteria [5].

Post-operative complications affect 30–40% of LT patients, with complication risks influenced by factors related to the donor (e.g., age, comorbidities, donor after cardiac death [DCD], cold ischemia time), recipient (e.g., age, comorbidities, model for end-stage liver disease [MELD], portal hypertension), and surgical aspects (e.g., portal thrombosis, anatomical variations, intraoperative complications).

The Comprehensive Complication Index (CCI^®^) was recently developed to assess cumulative complications following surgery [6]. This score has demonstrated high prognostic power across various clinical fields [7, 8]. CCI correlates with several indicators of poor post-surgical outcomes, underscoring its potential role in predicting early adverse outcomes. A Spanish study found that CCI correlated with frailty status in elderly patients undergoing surgery, suggesting a link between frailty, post-surgical complications, and poor outcomes [9].

A limited number of studies have examined the impact of post-operative complications following LT on HCC outcomes. A recent single-center retrospective study from Italy [10] reported a negative association between high CCI scores (< 47.3) and disease-free survival (DFS) (84% vs. 46%, *P* < 0.001), tumor-related death (3% vs. 26%, *P* < 0.001), and overall survival (OS) (89% vs. 62%, *P* < 0.001).

In our multi-institutional international study, we hypothesized that post-LT HCC-related mortality rates are higher in patients with a high CCI (≥ 42) measured at discharge compared to those with a lower CCI. We aimed to compare post-LT HCC-related mortality rates between two patient groups, categorized by high and low CCI following LT for HCC.

## Material and methods

### Study design

This retrospective, international multicenter observational study investigates the outcomes of patients undergoing LT for HCC treatment. Approval was obtained from the Local Ethics Board of Sapienza University of Rome, and the study followed the Strengthening the Reporting of Observational Studies in Epidemiology (STROBE) guidelines.

### Setting

Seven centers participated in this study: Ancona, Italy (*n* = 94); Brussels, Belgium (*n* = 157); Karolinska Institute, Sweden (*n* = 129); Padua, Italy (*n* = 309); Pisa, Italy (*n* = 307); Sapienza Rome, Italy (*n* = 121); and Udine, Italy (*n* = 143).

### Population

Between January 2005 and June 2019, data from 1260 patients were initially collected across the centers. Exclusion criteria included: (a) patient death within six months post-transplant (*n* = 127); (b) post-LT diagnosis of incidental HCC (*n* = 12); and (c) pediatric transplants (< 18 years) (*n* = 0). The final cohort comprised 1121 patients.

Patients were divided into two groups based on their CCI at discharge: Low-CCI Group (*n* = 942, 84.0%) and High-CCI Group (*n* = 179, 16.0%).

### Outcomes

The primary outcome was post-LT HCC-related mortality. Secondary outcomes included (a) overall patient survival, (b) overall HCC recurrence, and (c) early HCC recurrence (≤ 24 months post-LT). The last follow-up date was September 2021.

### Data collection

Data were extracted retrospectively from patient records. Data integrity was overseen by the study group’s Data Manager (QL), who resolved data errors and missing values through queries when feasible.

### Definitions

Complications were graded using the Dindo–Clavien Classification (CDC) [11]. CCI values were estimated using an online web calculator (available at https://www.assessurgery.com/), with the following original formula: CCI = [√(wC1 + wC2 … + wCx)]/2.

where each weighted complication (wC) corresponds to a specific Dindo–Clavien grade. CCI provides a cumulative score reflecting all complications (from 0, no complications, to 100, death).

EAD was defined according to the Olthoff criteria [12]. All complications were classified as previously described [13]. Organ procurement was categorized as “local” when performed in the same region as the LT.

### Statistical analysis

Continuous variables were presented as medians and interquartile ranges (IQR), while categorical variables were presented as counts and percentages. Categorical comparisons used Fisher’s exact test or chi-square test as appropriate, while the Mann–Whitney U test was used for continuous data. Missing data accounted for less than 10% of cases and were imputed using the median of nearby points due to the skewed distribution of variables.

The cohort was divided based on CCI values at discharge. Given the study’s non-randomized retrospective design, a stabilized inverse probability of treatment weighting (IPTW) was applied to balance the groups.

Propensity scores were calculated for each patient based on multivariable logistic regression, with HCC-related death post-LT as the dependent variable and 28 relevant confounders as covariates, including patient and donor demographics, liver disease etiology, MELD score, and HCC characteristics.

Stabilized weights were derived as follows:

SW = p/PS for the study group, and SW = (1 − p)/(1 − PS) for the control group, where *p* is the probability of etiology without considering covariates and PS is the propensity score.

Cohen’s D values were used to report effect sizes, with thresholds set as follows: |0.1| (very small), |0.1|–|0.3| (small), |0.3|–|0.5| (moderate), and >|0.5| (large).

Post-IPTW, multivariable logistic regression with mixed effects was conducted to identify risk factors for HCC-related death, overall death, overall recurrence, and early recurrence. The “LT Center” variable was included as a cluster-specific random effect. Significant variables were presented with odds ratios (OR) and 95% confidence intervals (CI). Kaplan–Meier survival analyses and the log-rank test were used for survival comparisons, with a statistical significance set at *P* < 0.05. SPSS version 27.0 was used for analysis.

## Results

A total of 1121 HCC patients met the study criteria and underwent LT at seven European centers between January 2005 and June 2019, with a median follow-up of 4.6 years (IQR = 3.1–6.9). Patient characteristics are detailed in Table 1.

**Table 1:** Characteristics of the investigated population (*n* = 1121)

Variables	Median (IQR) or n (%)		*P*-value
	Low-CCI (*n* = 942, 84.0%)	High-CCI (*n* = 179, 16.0%)	
*Patient*			
Age, years	59 (54–64)	59 (54–63)	0.88
Male sex	795 (84.4)	147 (82.1)	0.44
BMI	26 (24–28)	27 (24–30)	0.007
Waiting time length, months	3 (1–7)	4 (1–7)	0.27
Underlying liver disease			
HCV	535 (56.8)	99 (55.3)	0.74
HBV	224 (23.8)	30 (16.8)	0.04
Alcohol	235 (24.9)	55 (30.7)	0.11
NASH	62 (6.6)	11 (6.1)	1.00
Other	56 (5.9)	10 (5.6)	0.86
MELD	12 (9–16)	13 (10–22)	< 0.0001
*Donor*			
Age, years	63 (50–76)	60 (48–71)	0.02
Male sex	537 (57.0)	100 (55.9)	0.81
BMI	25 (23–28)	25 (23.28)	0.94
Cause of death			
CVA	641 (68.0)	129 (72.1)	0.33
Trauma	196 (20.8)	29 (16.2)	0.19
Anoxia	79 (8.4)	9 (5.0)	0.17
Other	39 (4.1)	15 (8.4)	0.02
ICU length of stay, days	3 (2–5)	3 (2–5)	0.48
Cardiac arrest	144 (15.3)	22 (12.3)	0.36
History of T2DM	99 (10.5)	14 (7.8)	0.34
Local procurement	531 (56.4)	108 (60.3)	0.37
*Transplant*			
CIT, minutes	42 (31–60)	42 (33–55)	< 0.0001
WIT, minutes	457 (393–517)	489 (415–565)	0.87
EAD	188 (20.0)	69 (38.5)	< 0.0001
ICU length of stay, days	4 (2–6)	6 (4–9)	< 0.0001
Hospital length of stay, days	14 (11–19)	23 (18–37)	< 0.0001
PNF	0 (-)	7 (3.9)	< 0.0001
HAT	13 (1.4)	12 (6.7)	< 0.0001
Biliary complication	206 (21.9)	72 (40.2)	< 0.0001
CCI	20.9 (0–26.2)	52.0 (45.4–61.3)	< 0.0001
*Tumor (anatomopathological assessment)*			
Diameter of the major lesion, cm	2.5 (1.5–3.5)	2.4 (1.4–3.5)	0.42
Number of nodules	2 (1–3)	2 (1–4)	0.69
Milan Criteria out status	328 (34.8)	69 (38.5)	0.35
Alpha-fetoprotein last available value, ng/mL	10 (4–47)	11 (4–56)	0.35
Poor grading (G3-4)	171 (18.2)	30 (16.8)	0.75
Microvascular invasion	269 (28.6)	44 (24.6)	0.32
Macrovascular invasion	33 (3.5)	10 (5.6)	0.20
RETREAT score	2 (1–5)	3 (1–4)	0.40

*CCI* comprehensive complication index; *n* number; *IQR* interquartile ranges; *BMI* body mass index; *HCV* hepatitis C virus; *HBV* hepatitis B virus; *NASH* non-alcoholic steatohepatitis; *MELD* model for end-stage liver disease; *CVA* cerebrovascular accident; *ICU* intensive care unit; *T2DM* type-2 diabetes mellitus; *CIT* cold ischemia time; *WIT* warm ischemia time; *EAD* early allograft dysfunction; *PNF* primary non-function; *HAT* hepatic artery thrombosis

There were no significant demographic differences between the Low-CCI and High-CCI groups regarding age, sex, waiting time, or underlying liver disease though High-CCI patients had higher median BMI (27 vs. 26; *P* = 0.007) and MELD scores (13 vs. 12; *P* < 0.0001).

Among donors, the High-CCI group had younger donors on average (median age: 60 vs. 63 years; *P* = 0.02). No significant differences were observed in donor sex, BMI, cause of death, ICU stay, history of type 2 diabetes, or cardiac arrest before organ procurement.

When comparing tumor characteristics, both groups had similar HCC morphology and poor grading, microvascular, and macrovascular invasion rates. However, the High-CCI group experienced more complications post-LT, resulting in a significantly higher median CCI score (52.0 vs. 20.9; *P* < 0.0001).

### Stabilized IPTW effect

To further reduce selection bias from the non-randomized study design, the groups were balanced using stabilized IPTW for 28 potential confounders (Table 2). Before IPTW, 18 variables showed very small differences, and ten showed small differences. After IPTW, 26 variables showed very small differences and only two showed small differences, with no change in the pseudo-population sample size.

**Table 2:** Effect of stabilized IPTW in the population on the variables used for balancing the two groups

Variables	Pre-IPTW			Post-IPTW		
	Mean (± SD)		Cohen’s *D*-value	Mean (± SD)		Cohen’s *D*-value
	Low-CCI (*n* = 942)	High-CCI (*n* = 179)		Low-CCI (*n* = 942)	High-CCI (*n* = 179)	
Patient age, years	58.1 ± 7.3	58.2 ± 7.1	− 0.01	58.0 ± 7.3	57.5 ± 7.3	0.08
Patient male sex	0.8 ± 0.4	0.8 ± 0.4	0.06	0.8 ± 0.4	0.8 ± 0.4	0.01
Patient BMI	26.2 ± 3.7	27.2 ± 4.5	− 0.22	26.4 ± 3.8	26.4 ± 4.2	− 0.01
Waiting time length, months	6.3 ± 11.2	6.4 ± 8.6	− 0.01	6.3 ± 11.1	6.4 ± 8.2	− 0.01
HCV	0.6 ± 0.5	0.6 ± 0.5	0.03	0.6 ± 0.5	0.6 ± 0.5	− 0.01
HBV	0.2 ± 0.4	0.2 ± 0.4	0.18	0.2 ± 0.4	0.2 ± 0.4	− 0.01
Alcohol	0.2 ± 0.4	0.3 ± 0.5	− 0.13	0.3 ± 0.4	0.3 ± 0.4	0.01
NASH	0.1 ± 0.2	0.1 ± 0.2	0.02	0.1 ± 0.2	0.1 ± 0.2	0.01
Other underlying liver disease	0.1 ± 0.2	0.1 ± 0.2	0.03	0.1 ± 0.2	0.0 ± 0.2	0.05
MELD	13.8 ± 7.0	16.0 ± 7.9	− 0.29	14.2 ± 7.4	14.7 ± 7.2	− 0.08
Donor age, years	61.4 ± 17.8	58.8 ± 15.7	0.16	60.9 ± 17.9	59.7 ± 15.9	0.07
Donor male sex	0.6 ± 0.5	0.6 ± 0.5	0.02	0.6 ± 0.5	0.6 ± 0.5	0.03
Donor BMI	25.7 ± 3.6	25.7 ± 4.1	0.00	25.7 ± 3.6	25.9 ± 4.1	− 0.04
CVA as donor cause of death	0.7 ± 0.5	0.7 ± 0.4	− 0.09	0.7 ± 0.5	0.7 ± 0.5	− 0.01
Trauma as donor cause of death	0.2 ± 0.4	0.2 ± 0.4	0.12	0.2 ± 0.4	0.2 ± 0.4	− 0.03
Anoxia as donor cause of death	0.1 ± 0.3	0.1 ± 0.2	0.15	0.1 ± 0.3	0.1 ± 0.3	0.03
Other donor cause of death	0.0 ± 0.2	0.1 ± 0.3	− 0.16	0.0 ± 0.2	0.1 ± 0.2	− 0.02
Donor ICU length of stay, days	4.4 ± 4.1	4.2 ± 3.7	0.06	4.3 ± 4.1	4.3 ± 3.9	0.01
Donor cardiac arrest	0.2 ± 0.4	0.1 ± 0.3	0.09	0.2 ± 0.4	0.1 ± 0.3	0.08
Donor history of T2DM	0.1 ± 0.3	0.1 ± 0.3	0.10	0.1 ± 0.3	0.1 ± 0.3	0.06
Donor local procurement	0.6 ± 0.5	0.6 ± 0.5	− 0.08	0.6 ± 0.5	0.6 ± 0.5	− 0.10
CIT, minutes	462.1 ± 118.0	492.5 ± 129.7	− 0.24	467.3 ± 122.0	470.0 ± 118.5	− 0.02
WIT, minutes	46.6 ± 20.2	46.2 ± 18.9	0.02	46.5 ± 20.1	46.6 ± 19.1	0.00
Diameter of the HCC major lesion, cm	2.7 ± 2.2	2.6 ± 2.2	0.01	2.6 ± 2.2	2.7 ± 2.2	− 0.04
Number of HCC nodules	2.8 ± 3.3	2.7 ± 3.1	0.03	2.8 ± 3.4	2.7 ± 3.0	0.03
Poor grading (G3–4)	0.2 ± 0.4	0.2 ± 0.4	0.04	0.2 ± 0.4	0.2 ± 0.4	0.03
Microvascular invasion	0.3 ± 0.5	0.2 ± 0.4	0.09	0.3 ± 0.5	0.3 ± 0.4	0.06
Macrovascular invasion	0.0 ± 0.2	0.1 ± 0.2	− 0.09	0.0 ± 0.2	0.1 ± 0.2	− 0.10

*IPTW* inverse probability therapy weighting; *CCI* comprehensive complication index; *n* number; *SD* standard deviation; *BMI* body mass index; *HCV* hepatitis C virus; *HBV* hepatitis B virus; *NASH* non-alcoholic steatohepatitis; *MELD* model for end-stage liver disease; *CVA* cerebrovascular accident; *ICU* intensive care unit; *T2DM* type-2 diabetes mellitus; *CIT* cold ischemia time; *WIT* warm ischemia time

### Risk factors for death and recurrence rates

In the post-IPTW cohort, four mixed effects multivariable logistic regression models identified risk factors for HCC-related death, overall death, overall recurrence, and early recurrence, as shown in Table 3. Tumor-related covariates (e.g., tumor diameter, nodule count, and microvascular invasion) were significant in all models. A CCI score of ≥ 42 was also a relevant independent risk factor across all models. In detail, CCI ≥ 42 increased the odds by 3.35 times (OR = 3.35; *P* < 0.0001) for the risk of HCC-related death; by 2.63 times (OR = 2.63; *P* < 0.0001) for the risk of overall death; by 2.09 times (OR = 2.09; *P* = 0.001) for the risk of overall recurrence; and by 1.88 times (OR = 1.88; *P* = 0.02) for the risk of early recurrence.

**Table 3:** Multivariable logistic regression models for the risks of HCC-related death, overall death, overall HCC recurrence, and early HCC recurrence

Variables	Beta	SE	Wald	OR	95.0%CI		*P*-value
					Lower	Upper	
*a) HCC-related death*							
CCI ≥ 42	1.21	0.26	21.81	3.35	2.02	5.57	< 0.0001
Microvascular invasion	1.03	0.23	19.52	2.80	1.78	4.43	< 0.0001
Number of HCC nodules	0.10	0.08	14.45	1.11	1.05	1.17	< 0.0001
Diameter of the HCC major lesion, cm	0.15	0.04	14.34	1.16	1.07	1.25	< 0.0001
HCV	0.63	0.24	6.94	1.88	1.18	2.99	0.008
MELD	− 0.04	0.02	5.40	0.96	0.93	0.99	0.02
*b) overall death*							
CCI ≥ 42	0.99	0.19	26.89	2.63	1.83	3.80	< 0.0001
HCV	0.48	0.17	7.51	1.61	1.15	2.26	0.006
Microvascular invasion	0.87	0.34	6.65	2.38	1.23	4.62	0.01
Number of HCC nodules	0.06	0.02	6.58	1.06	1.01	1.10	0.01
Diameter of the HCC major lesion, cm	0.08	0.03	5.50	1.08	1.01	1.15	0.02
Donor local procurement	− 0.37	0.16	5.31	0.69	0.51	0.95	0.02
Alcohol-related cirrhosis	0.41	0.19	4.63	1.50	1.04	2.17	0.03
CIT, minutes	0.001	0.00	3.89	1.001	1.000	1.002	0.049
*c) overall HCC recurrence*							
Microvascular invasion	0.995	0.19	26.58	2.71	1.85	3.95	< 0.0001
Number of HCC nodules	0.12	0.02	23.27	1.13	1.07	1.18	< 0.0001
Diameter of the HCC major lesion, cm	0.13	0.04	13.14	1.14	1.06	1.23	< 0.0001
CCI ≥ 42	0.74	0.23	10.09	2.09	1.33	3.29	0.001
MELD	− 0.03	0.02	5.08	0.97	0.94	0.996	0.02
HCV	0.42	0.20	4.59	1.52	1.04	2.24	0.03
CIT, minutes	0.002	0.001	4.24	1.002	1.000	1.003	0.04
*d) early (*≤ *24 months) HCC recurrence*							
Microvascular invasion	1.26	0.23	31.19	3.52	2.27	5.48	< 0.0001
Number of HCC nodules	0.11	0.03	16.76	1.11	1.06	1.17	< 0.0001
Diameter of the HCC major lesion, cm	0.14	0.04	14.01	1.15	1.07	1.24	< 0.0001
Patient BMI	− 0.09	0.03	8.47	0.91	0.86	0.97	0.004
Anoxia as cause of donor death	0.84	0.35	5.70	2.31	1.16	4.59	0.02
CCI ≥ 42	0.63	0.27	5.34	1.88	1.10	3.20	0.02

− 2Log likelihood: a) 566.2; b) 1061.1; c) 759.2; d) 581.8

“LT Center” variable incorporated in all the models as a cluster-specific random effect variable

Variable initially introduced in all the models: era of LT (2005–10 vs. 2011–19), patient age, patient male sex, patient BMI, waiting time length, HCV, HBV, alcohol, NASH, other underlying liver disease, MELD, donor age, donor male sex, donor BMI, CVA as donor cause of death, trauma as donor cause of death, anoxia as donor cause of death, other donor cause of death, donor ICU length of stay, donor cardiac arrest, donor history of T2DM, donor local procurement, CIT, WIT, diameter of the HCC major lesion, number of HCC nodules, poor grading, microvascular invasion, macrovascular invasion

*SE* standard error; *OR* odds ratio; *CI* confidence intervals; *HCC* hepatocellular cancer; *CCI* comprehensive complication index; *HCV* hepatitis C virus; *MELD* model for end-stage liver disease; *CIT* cold ischemia time; *MELD* model for end-stage liver disease; *BMI* body mass index; *LT* liver transplantation; *HBV* hepatitis B virus; *NASH* non-alcoholic steatohepatitis; *CVA* cerebrovascular accident; *ICU* intensive care unit; *T2DM* type-2 diabetes mellitus; *WIT* warm ischemia time

### Post-transplant survival rates

**Fig. 1:**
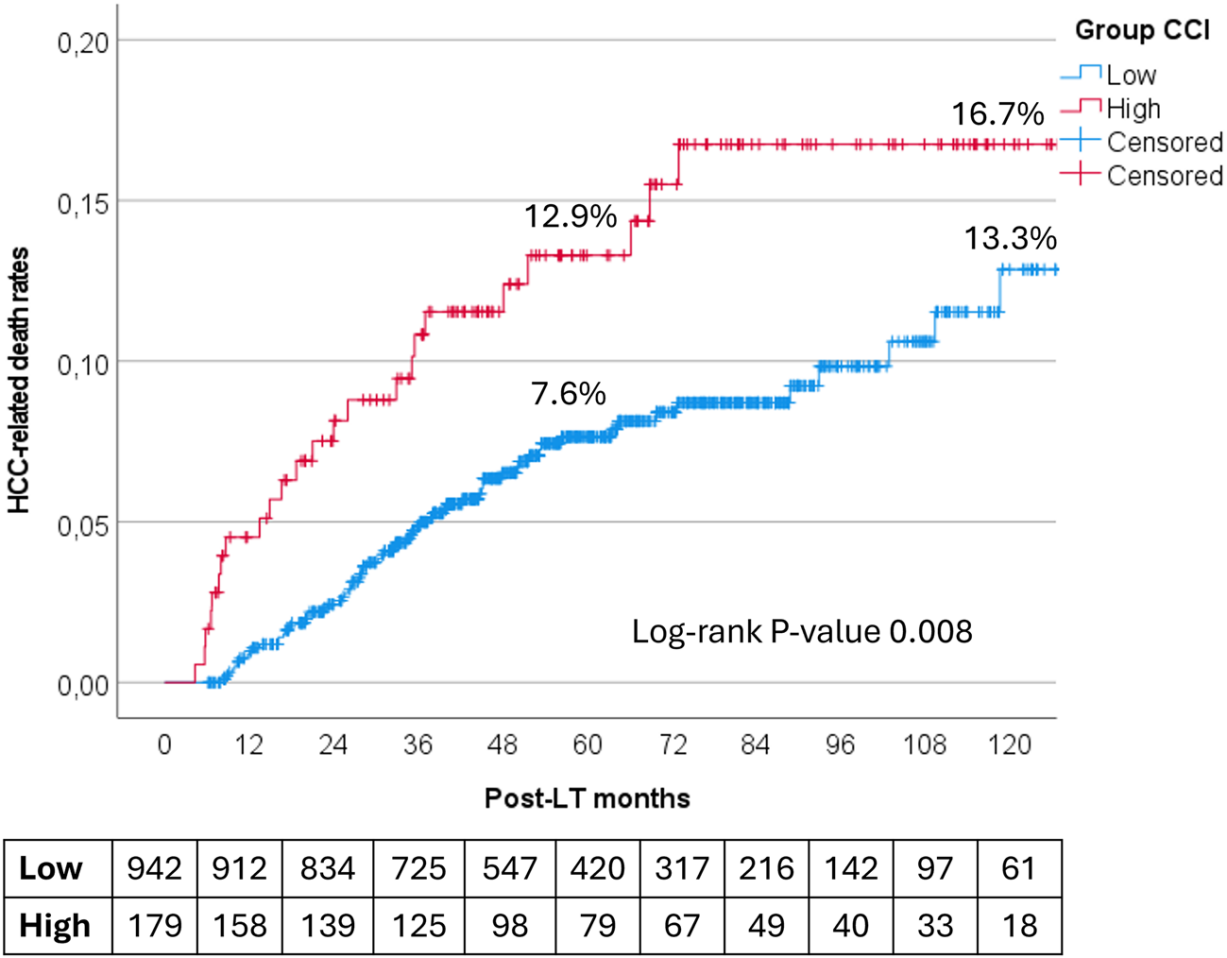
HCC-related death rates in patients with high (≥ 42) versus low (< 42) CCI

In the post-IPTW cohort, during the follow-up period, 225 of the 1121 patients (20.1%) died, with 92 deaths (8.2%) attributed to HCC-related causes. The 5- and 10-year overall survival rates were 84.1% and 74.4%, respectively, for Low-CCI patients and 68.2% and 52.4% for High-CCI patients (*P* < 0.0001). The 5- and 10-year HCC-related mortality rates were 7.6% and 13.3% for Low-CCI patients and 12.9% and 16.7% for High-CCI patients (*P* = 0.008) (Fig. 1).

**Fig. 2:**
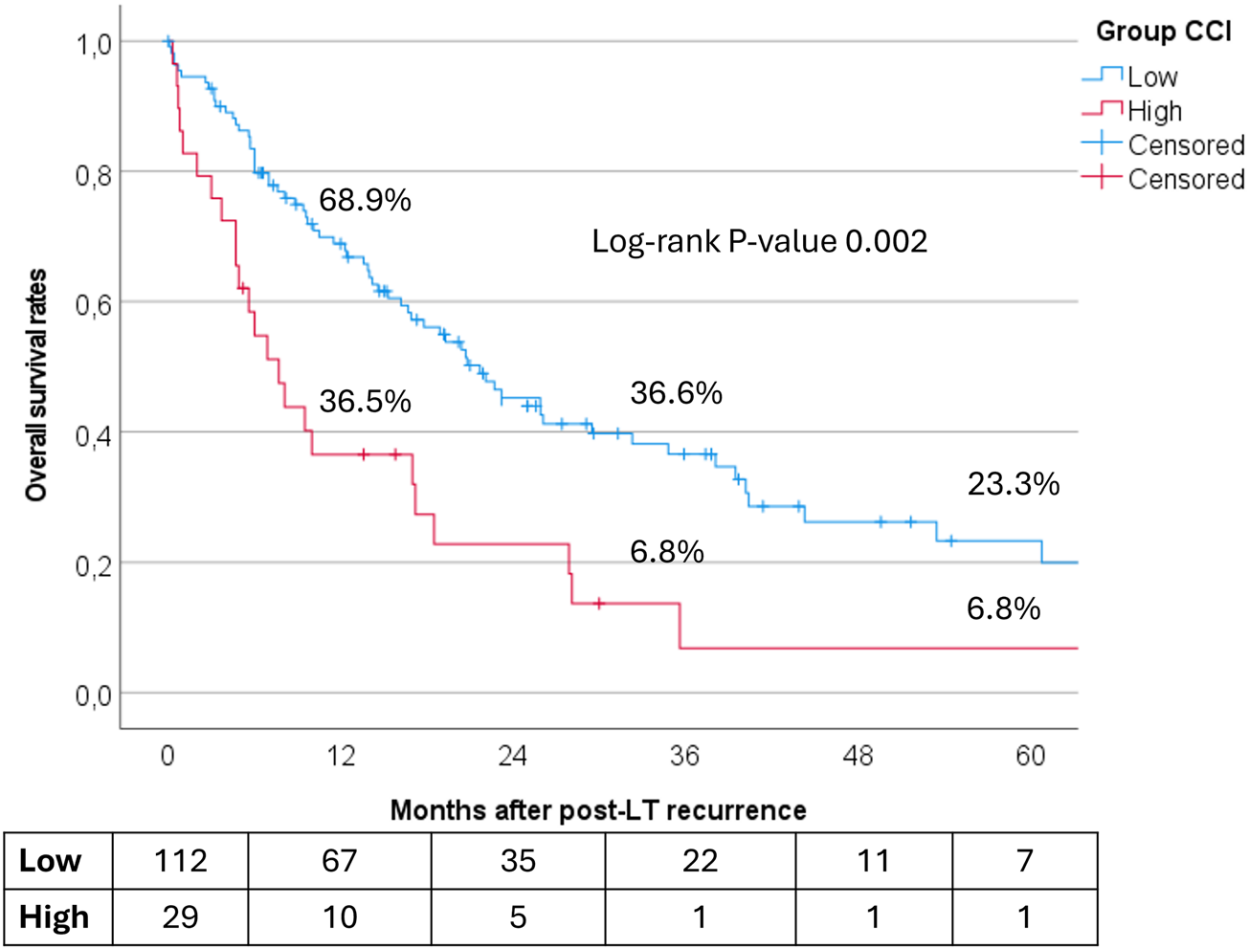
Overall survival rates in recurred patients with high (≥ 42) versus low (< 42) CCI

In total, 141 of the 1121 patients (12.6%) experienced HCC recurrence, with 99 cases (8.8%) occurring within 24 months post-LT. The 5- and 10-year recurrence rates were 12.0% and 17.5%, respectively, for Low-CCI patients and 15.0% and 19.1% for High-CCI patients (*P* = 0.11). Following recurrence, Low-CCI patients showed better outcomes, with 3- and 5-year survival rates of 36.6% and 23.3%, compared to 6.8% for both 3- and 5-year rates in High-CCI patients (*P* = 0.002) (Fig. 2).

## Discussion

This study shows that major complications following LT significantly impair HCC outcomes. Specifically, a CCI score > 42 at discharge is a strong prognostic factor for HCC-related mortality, overall recurrence, and early recurrence, comparable to key tumor-related characteristics. Moreover, recurred patients with a high CCI show worse post-recurrence survivals, denoting a more aggressive recurrence in patients with a higher CCI value at LT discharge.

The CCI was initially developed to enhance the Dindo–Clavien Classification by providing a cumulative assessment of morbidity through weighted complications [6]. Multiple studies have highlighted the prognostic value of CCI in oncology settings. For instance, a study in Japan linked post-operative complications to poorer survival in gastric cancer patients, finding significantly lower 5-year overall and disease-specific survival among patients with CCI ≥ 32.15. The study also identified CCI as an independent predictor of poor prognosis through multivariable analysis [14].

In a U.S.-based study, CCI was a significant predictor of survival in patients undergoing liver resection for colorectal metastases, independent of RAS mutation status. Patients with CCI ≥ 26.2 had worse recurrence-free and cancer-specific survival rates compared to those with lower CCI scores [7]. For HCC, CCI outperformed the Dindo–Clavien Classification in predicting length of hospital stay following hepatic resection [15]. A recent study by Hendo et al. [16] involving 1124 HCC patients who underwent hepatic resection with favorable tumor characteristics (low-to-medium AFP levels and tumor burden scores) found that moderate-to-severe complications adversely impacted prognosis.

Our team previously presented data on 1782 LT patients, demonstrating that CCI is a reliable predictor of 90-day and 1-year graft loss across various patient subgroups, with better diagnostic accuracy than other pre- and post-LT scores [13]. Additionally, a Dutch study investigating the relationship between CCI and biliary complications compared transplants using DCD and donation after brain death (DBD) grafts. The six-month post-operative CCI median value was significantly higher in DCD recipients (53.4 vs. 47.2), along with a higher rate of re-transplantation due to ischemic cholangiopathy lesions (4% vs. 1%) [17].

Few studies have examined the link between CCI and HCC recurrence risk post-transplant. A single-center retrospective analysis from the Milan Niguarda Hospital reported that post-operative complications were associated with long-term survival and recurrence outcomes, using a CCI threshold of < 47.3 [10].

Our findings confirm these results and provide additional insights. This is the first multi-institutional, international study investigating CCI role in HCC outcomes, covering high-, medium-, and low-volume centers across Europe and demonstrating that high complication rates have consistent adverse effects regardless of institutional volume.

Apart from the studies investigating the role of CCI, several articles have explored the correlation between surgical complications and HCC recurrence, mainly in the case of hepatic resection [18–20]. In detail, a study from China (*n* = 393) reported that a Dindo–Clavien Grades II–IV correlated with an increased risk for advanced post-resection HCC recurrence (OR = 6.25, 95%CI = 1.72–22.66; *P*-value = 0.005) [18].

A study from Japan (*n* = 475) explored the predictors of early (< 2-year) recurrence after resection, showing that Dindo–Clavien Grades III–IV correlated with this risk (OR = 1.72, 95%CI = 1.05–2.85: *P*-value = 0.032) [19]. Another study from Japan (*n* = 145) specifically evaluated the impact of post-surgical complications on early-phase recurrence after HCC curative resection, observing that early recurrence was observed in 53% versus 34% of patients with versus without post-surgical complications (*P*-value = 0.039) [20].

A significant strength of the present study is the statistical approach used to minimize confounding. By employing stabilized IPTW, we balanced the population, reducing potential bias from confounding variables. Notably, the median donor age in the High-CCI group was lower than that in the Low-CCI group, indicating that older donors do not necessarily contribute to poorer outcomes. Moreover, early allograft dysfunction (EAD) incidence was higher in the Low-CCI group. EAD generally correlates with longer ICU or hospital stays and a higher rate of post-LT complications. Our findings align with a recent review by Wehrle et al., which noted that EAD clinical presentation and management are not yet fully captured by the CCI score [21].

These findings could aid in managing HCC patients at discharge and during follow-up, promoting a personalized immunosuppression regimen (e.g., early introduction of everolimus, steroid withdrawal, and calcineurin minimization) and a structured follow-up schedule with regular AFP and PIVKA testing and stricter imaging protocols. Moreover, it is intriguing to consider the possibility to integrate the early post-LT clinical course into already adopted scores of prognostications for the risk of recurrence, like the RETREAT or the RELAPSE scores [5, 22]. Another relevant point to consider is that recurred patients with a high CCI present a more aggressive clinical course, with poor long-term overall survival rates. Therefore, this finding may suggest selecting more aggressive therapeutic strategies in this specific setting. However, further studies are needed to investigate the relationship between CCI and HCC in greater detail to clarify these relevant aspects.

Apart from the potential usefulness of the CCI in the post-operative course, it is relevant to underline that the minimization of an adverse post-LT surgical course is mandatory in HCC patients with the intent to positively impact their oncological story. Therefore, careful management is essential in the pre-transplant phase. HCC patients are often transplanted using extended criteria donors (ECD) or DCD grafts, which are more vulnerable to ischemia–reperfusion injury (IRI) and tend to result in poorer clinical outcomes post-LT. Studies have explored the association between graft quality and HCC recurrence [23, 24]. Strategies to mitigate IRI are critical, and machine perfusion has shown promise in preserving ECD grafts, leading to reduced post-LT complication rates [25, 26].

Our study has some limitations. As a retrospective, multicenter study, there is a possibility of missing data, particularly for lower-grade complications (grades I–II). The retrospective collection of all pharmacological needs post-LT poses challenges, yet despite the possibility of underreporting, CCI demonstrated high prognostic value in our dataset, suggesting its potential as an even stronger predictor with a prospectively collected database.

The study spanned over 14 years, during which advancements in surgical techniques, post-operative care, and immunosuppressive therapies may have influenced outcomes. Differences in practices over time could lead to heterogeneity in patient management and outcomes that are not fully accounted for in the analysis.

Another limitation is the variability in CCI grading for certain complications due to the lack of a standardized literature basis, which may impact the score’s performance.

Although the IPTW approach helped balance the study groups, residual confounding may persist, particularly if certain unmeasured variables influenced group assignment. This potential bias could limit the accuracy of the observed relationships between CCI and outcomes.

Another limit is the decision of the adopted CCI threshold. Different studies have reported several cut-offs, with no specific identification of an internationally recognized threshold value. We adopted the value identified in our previous study [13]. However, we noted that the CCI cut-off in the setting of LT was very close among the studies published, ranging 42–47 [10, 13].

Lastly, given that the study focus on European centers, findings may not fully generalize to populations outside of Europe. Differences in donor availability, liver disease etiology, and transplant practices between regions could affect the applicability of the study’s conclusions to other healthcare settings, such as North America or Asia.

## Conclusions

In conclusion, our study highlights that high-grade in-hospital complications post-LT adversely affect oncologic outcomes in HCC patients. Further research is necessary to validate these findings and to develop a prognostic score incorporating post-operative complications.

## Supplementary Information

Below is the link to the electronic supplementary material.

Supplementary file1 (DOCX 19 KB)

## Data Availability

The datasets generated during and/or analyzed during the current study are not publicly available but are available from the corresponding author on reasonable request.

## Abbreviations

AFP: Alpha-fetoprotein
AI: Artificial intelligence
BMI: Body mass index
CCI: Comprehensive complication index
CDC: Dindo–Clavien classification
CIT: Cold ischemia time
CVA: Cerebrovascular accident
DBD: Donation after brain death
DCD: Donation after cardiac death
DFS: Disease-free survival
EAD: Early allograft dysfunction
ECD: Extended criteria donors
HAT: Hepatic artery thrombosis
HCC: Hepatocellular carcinoma
HBV: Hepatitis B virus
HCV: Hepatitis C virus
ICU: Intensive care unit
IPTW: Inverse probability of treatment weighting
IQR: Interquartile range
LT: Liver transplantation
MELD: Model for end-stage liver disease
NASH: Non-alcoholic steatohepatitis
OS: Overall survival
PIVKA II: Protein induced by vitamin K absence or antagonist-II
PNF: Primary non-function
RETREAT: Risk estimation of tumor recurrence after transplant
SE: Standard error
STROBE: Strengthening the reporting of observational studies in epidemiology
T2DM: Type-2 diabetes mellitus
WIT: Warm ischemia time
