# Supplementary material for: The role of the comprehensive complication index in the prediction of tumor-related death in transplanted patients with hepatocellular carcinoma
Source: Updates Surg. 2025 Feb 10;77(3):705–15. doi: 10.1007/s13304-025-02101-8 (PMC12226674; doi:10.1007/s13304-025-02101-8)
Supplement: Supplementary file 1 — Supplementary file1 (DOCX 19 KB) [file 13304_2025_2101_MOESM1_ESM.docx]

**Supplementary data**

**Supplementary Table 1.** Missing data in the initial population of 1,260 cases (before removing exclusion criteria cases).

| **Variables** | **Missing cases** | **%** |
| --- | --- | --- |
| Patient age | 0 | 0.0 |
| Patient sex | 1 | 0.1 |
| BMI | 33 | 2.6 |
| Underlying liver disease | 0 | 0.0 |
| MELD | 0 | 0.0 |
| Donor age | 7 | 0.6 |
| Donor sex | 42 | 3.3 |
| Donor BMI | 23 | 1.8 |
| Cause of donor death | 20 | 1.6 |
| Donor ICU lenght of stay | 40 | 3.2 |
| Donor cardiac arrest | 33 | 2.6 |
| Donor T2DM | 36 | 2.9 |
| Local organ procurement | 5 | 0.4 |
| CIT | 13 | 1.0 |
| WIT | 29 | 2.3 |
| EAD Olthoff | 13 | 1.0 |
| Patient ICU lenght of stay | 101 | 8.0 |
| Patient lenght of stay | 13 | 1.0 |
| PNF | 0 | 0.0 |
| HAT | 17 | 1.3 |
| Biliary complications | 13 | 1.0 |
| Re-transplantation | 0 | 0.0 |
| CCI points | 0 | 0.0 |
| Waiting time, months | 27 | 2.1 |
| Diameter of the major HCC lesion | 19 | 1.5 |
| Number of lesions | 23 | 1.8 |
| Grading | 36 | 2.9 |
| Microvascular invasion | 39 | 3.1 |
| Macrovascular invasion | 41 | 3.3 |
| Death | 0 | 0.0 |
| Graft loss | 0 | 0.0 |
| HCC recurrence | 0 | 0.0 |
| **Abbreviations:** %, percentage; BMI, body mass index; MELD, model for end-stage liver disease; ICU, intensive care unit; T2DM, type-2 diabetes mellitus; CIT, cold ischemia time; WIT, warm ischemia time; EAD, early allograft dysfunction; PNF, primary non-function; HAT, hepatic artery thrombosis; CCI, comprehensive complication index; HCC, hepatocellular cancer. | | |
